# Spillover infections by rustrela virus, borna disease virus 1 and tick-borne encephalitis virus revealed by retrospective screening of mammalian encephalitis of unknown origin

**DOI:** 10.1186/s12917-025-05132-w

**Published:** 2025-11-15

**Authors:** Anne Voss, Anne Günther, Olga Geit, Chloé Puget, Andreas Pauly, Karin Stiasny, Kore Schlottau, Martin Beer, Achim D. Gruber, Angele Breithaupt, Dennis Rubbenstroth, Lars Mundhenk

**Affiliations:** 1 Institute of Veterinary Pathology, Freie Universität Berlin, Robert-von-Ostertag-Straße 15, 14163 Berlin, Germany; 2 Tierpark Berlin-Friedrichsfelde GmbH, Am Tierpark 125, 10319 Berlin, Germany; 3 Center for Virology, Medical University of Vienna, Kinderspitalgasse 15, 1090 Vienna, Austria; 4 Institute of Diagnostic Virology, Friedrich-Loeffler-Institut, Südufer 10, 17493 Greifswald - Insel Riems, Germany; 5 Department of Experimental Animal Facilities and Biorisk Management, Friedrich- Loeffler-Institut, Südufer 10, 17493 Greifswald - Insel Riems, Germany

**Keywords:** RusV, BoDV-1, TBEV, Influenza a virus (IAV), HPAIV H5, Meningoencephalitis

## Abstract

**Background:**

Cross-species transmission of several viral neuropathogens may lead to fatal disease in incidental hosts. The newly discovered rustrela virus (RusV) as well as Borna disease virus 1 (BoDV-1), tick-borne encephalitis virus (TBEV), and highly pathogenic avian influenza virus (HPAIV) of hemagglutinin subtype H5 may cause fatal lymphocytic meningoencephalitis in a broad range of mammalian species after crossing species borders. Here, we tested brain tissue samples from 191 animals representing 19 mammalian species diagnosed with lymphocytic meningoencephalitis from 1989 to 2024 for these four neuropathogens by RT-qPCR. Positive samples were analysed for cell-associated viral RNA or viral antigen by RNA in situ hybridisation or immunohistochemistry, respectively.

**Results:**

For the first time RusV was detected in one out of two tested maned wolves (50%). Further, two out of 50 cats (4.0%) and the only tested donkey were infected. BoDV-1 and TBEV were found in three out of eight horses (37.5%) and one out of 78 dogs (1.3%), respectively. Neurons were the main target cells for all three pathogens. Partial genomic RusV and BoDV-1 sequences matched with the predominant virus types in the study region. Influenza A virus RNA was not detected in any of the samples.

**Conclusions:**

The host range of RusV was extended to Canidae, represented by a fatal case of a maned wolf. Both RusV and BoDV-1 seem to be important pathogens causing lymphocytic meningoencephalitis in other mammalian species and their distribution should be monitored closely.

**Supplementary Information:**

The online version contains supplementary material available at 10.1186/s12917-025-05132-w.

## Background

Crossing host species barriers by numerous pathogens, termed spillover infections, is now considered not only the cause of epidemiologically relevant pandemics (e.g. SARS-CoV2 [1], canine parvovirus 2 [2]), but also of sporadic dead-end host infections that may lead to fatal outcome in individuals only without further transmission. For example, various neurotropic viruses have been shown to cross mammalian species barriers [3], including rustrela virus (RusV; *Rubivirus strelense*), Borna disease virus 1 (BoDV-1; *Orthobornavirus bornaense*), tick-borne encephalitis virus (TBEV; *Orthoflavivirus encephalitidis*) and highly pathogenic avian influenza virus of hemagglutinin subtype H5 (HPAIV H5; *Alphainfluenzavirus influenzae*) [4–17]. Common to all these viruses is a rather stereotypic immune host response, typically lymphocytic meningoencephalitis. This inflammatory pattern is highly unspecific from the histopathology perspective during routine post mortem examinations [4–17]. Therefore, we speculated that spillover cases involving these viruses may have remained undetected in the past.

RusV is a recently discovered neurotropic pathogen that belongs to the family *Matonaviridae* and is closely related to rubella virus (*Rubivirus rubellae*) causing “German measles” in humans, which had long been considered the only known representative of the genus *Rubivirus* [4]. RusV was initially identified in zoo and wildlife animals with fatal meningoencephalitis in northern Germany, including a donkey (*Equus asinus*), a capybara (*Hydrochoerus hydrochaeris*), several red-necked wallabies (*Macropus rufogriseus*) [4, 5, 18], a South American coati (*Nasua nasua*), an Eurasian otter (*Lutra lutra*) [7], and numerous lions (*Panthera leo*) [19, 20]. Furthermore, the virus was also detected in a wild mountain lion (*Puma concolor*) in the USA [21]. Moreover, RusV was also found in domestic cats (*Felis catus*) associated with a lethal inflammatory neurological disorder, termed “staggering disease” [6, 22, 23]. In all affected animals, the virus seems to be strictly neurotropic [4, 7, 18]. It is assumed that RusV infections originate from a rodent reservoir, since RusV was also detected in apparently non-encephalitic yellow-necked field mice (*Apodemus flavicollis*) in Germany and wood mice (*Apodemus sylvaticus*) in Sweden [4, 6, 7, 24].

BoDV-1 is the etiologic agent of Borna disease (BD), a fatal neurological disease in humans and domestic animals, in particular horses, sheep, and New World camelids [8, 10]. Spillover infections from the reservoir host, the bicoloured white-toothed shrew (*Crocidura leucodon*) [10, 25–27] to spillover hosts cause T lymphocyte-mediated meningoencephalitis with a strict neurotropism of the virus [28–30]. The virus has been demonstrated only in endemic areas in Germany, Switzerland, Austria, and Liechtenstein [8, 10, 11, 31, 32].

TBEV, which is endemic in different regions of central Europe, Siberia, and Southeast Asia, causes tick-borne encephalitis (TBE), a lymphocytic meningoencephalitis in humans [33]. In addition to humans, the virus has also been sporadically detected in dogs, horses, domestic and wild ruminants with different clinical manifestations [9, 12, 34–37]. Small mammals, such as bank voles (*Myodes glareolus*) and yellow-necked field mice, are suspected to be the main reservoirs of the virus, which is primarily transmitted to accidental hosts via ticks, particularly the sheep tick (*Ixodes ricinus*) [38].

The genetic descendants of the HPAIV H5 goose/Guangdong lineage (gs/GD; clade 2.3.4.4b), whose common ancestor emerged almost 30 years ago in Southeast Asia, are currently held responsible for an almost global panzootic. Although still primarily associated with cases in mainly aquatic wild birds and outbreaks in domestic poultry, recent trends reveal a notable increase of HPAIV H5 infections in mammals [39, 40]. The virus may cause lymphocytic and necrotizing meningoencephalitis in a wide range of carnivores, such as red foxes, mustelids, felids, seals, and other mammals, with consumption of infected wild bird carcasses assumed to be the predominant transmission route [13–15, 17, 39].

Here, we tested for possible previous spillover events of these viral neuropathogens in a broad range of mammalian species. To this end, archived brain tissues from 191 animals representing 19 different species diagnosed with lymphocytic meningoencephalitis of unknown aetiology were retrospectively analysed covering a period of 36 years.

## Materials and methods

### Case selection

The diagnostic archive of the Institute of Veterinary Pathology of Freie Universität Berlin was screened for cases of lymphocytic meningoencephalitis of unknown aetiology during the period of 1989 and 2023. The database was first screened for the terms encephalitis, meningoencephalitis, or meningitis. Subsequently, cases with suppurative inflammation as well as cases with an already established etiological diagnosis such as infections with canine distemper virus, feline mutated coronavirus (feline infectious peritonitis), herpesviruses, small ruminant lentiviruses, *Toxoplasma gondii* and *Neospora caninum* were excluded. Animals necropsied at the institute for which RusV or BoDV-1 infection had already been published in previous studies were excluded [6, 8, 10, 18, 28]. A maned wolf with neurological signs, examined in 2024, and diagnosed with a lymphocytic meningoencephalitis was additionally tested for the four viruses. In total, 191 cases were included, representing 19 different species of livestock, pets, zoo and wild animals. The majority of animals were dogs (*Canis lupus familiaris;* *n* = 78, 40.8%) and cats (*Felis catus*; *n* = 50; 26.2%), followed by cattle (*Bos taurus;* *n* = 12, 6.3%), goats (*Capra aegagrus hircus*; *n* = 11, 5.8%), horses (*Equus caballus*; *n* = 8, 4.1%), pigs (*Sus scrofa domesticus*; *n* = 7, 3.7%), sheep (*Ovis gmelini aries*; *n* = 6, 3.1%), and guinea pigs (*Cavia porcellus*; *n* = 6, 3.1%). Only one to two animals were analysed for 11 further species (Table 1).

A two-step strategy for viral detection was performed: first, all cases were screened for RNA of the respective viruses using reverse transcription quantitative PCR (RT-qPCR). Cases tested positive by PCR were further analysed using RNA in situ hybridisation (ISH) or immunohistochemistry (IHC).

### RNA extraction

Total RNA was extracted from three 5 μm scrolls of one formalin-fixed, paraffin-embedded (FFPE) tissue block containing cerebrum, cerebellum, or brainstem using the miRNAeasy FFPE Kit (QIAGEN, USA). In brief, samples were deparaffinised by adding 320 µl deparaffinization solution (QIAGEN, USA) and incubation for 3 min at 56 °C. All further steps were performed according to the manufacturer’s protocol. Finally, RNA was eluted with 20 µl RNase free water. For the maned wolf, total RNA was obtained from fresh frozen material, as described previously [6].

### RT-qPCR analysis

Samples were tested for viral RNA by specific RT-qPCR assays. All primer and probe sequences as well as RT-qPCR protocols are listed in Additional file 1: Table S1. RusV RNA was detected by a two-step RT-qPCR protocol modified from the one-step panRusV-2a assay [23], with a separate reverse transcription step. cDNA was synthesised from total RNA using the iScript cDNA Synthesis Kit (BioRad, USA) according to the manufacturer’s protocol. Subsequently, qPCR was performed with the Maxima Probe qPCR Master Mix Kit (ThermoFisher Scientific, USA) according to manufacturer’s instructions. Samples were classified as positive when both technical replicates exhibited a cycle of quantification (Cq) value of less than 40. Non-template controls failed to show any Cq value. A RT-qPCR for the housekeeping gene eukaryotic translation elongation factor 1a (EF1a) tested for RNA extraction efficacy and quality, as previously published [41].

Testing for BoDV-1- and TBEV-specific RNA was performed in a one-step approach including reverse transcription (cycler settings displayed in Additional file 1: Table S1). BoDV-1 detection was performed as previously described [42] by detecting the BoDV-1 phosphoprotein (P) gene using the qScript XLT One-Step RT-qPCR ToughMix (QuantaBio, USA). To control for RNA quality, a duplex assay with a primer-probe-system targeting the beta-actin (ACTB) gene [43] was utilised.

A duplex assay targeting TBEV and an internal control was performed employing AgPath-ID One-Step RT-PCR Reagents (Ambion, USA), as previously described [44]. As an internal control, a predetermined copy number of in vitro-transcribed RNA of the enhanced green fluorescent protein (eGFP) gene [45] was added to the RT-qPCR master mix.

RT-qPCR for influenza A virus (IAV) RNA aimed for the detection of the matrix (M) gene. The master mix was prepared with AgPath-ID One-Step RT-PCR Reagents (Ambion, USA) complemented by a primer-probe-combination, as described by Hassan at el [46].

For validation of the assay and calibration of the Cq values, a suitable positive control RNA was included in each run.

### Partial genome sequencing of RusV and BoDV-1

Generation of partial RusV p150-encoding sequences was performed for all RusV-positive animals by Sanger sequencing of four overlapping conventional RT-PCR amplicons of 142 to 191 base pairs in length, as described by de le Roi et al. [19] to achieve a sequence of 409 nucleotide (nt) length, representing genome positions 100 to 508 of RusV reference genome MN552442.2. For all BoDV-1-positive animals, sequencing of the nucleoprotein (N), accessory protein (X), and phosphoprotein (P) genes (1,824 nt; positions 54 to 1,877 of reference genome U04608.1) was attempted by sequencing of overlapping amplicons of ~ 150 bp length. The same strategy was attempted to determine partial TBEV envelope protein (E) gene sequences from TBEV-positive samples. Primer sequences are available upon request. Each amplicon was sequenced in both directions and raw sequences were trimmed for quality and primer sequences before assembly. Sequences generated in this study are deposited in GenBank under accession numbers PV806686 to PV806690.

Phylogenetic analysis of these sequences was performed together with all RusV (*n* = 74) or BoDV-1 (*n* = 259) sequences derived from GenBank for which the respective sequence stretch was available. The nucleotide sequences were aligned using MUSCLE 3.8.425 (available in Geneious Prime 2021.0.1, Auckland, New Zealand), before a Neighbour-Joining (NJ) consensus tree was calculated with 1,000 bootstrap replicates.

### RNA in situ hybridization and immunohistochemistry for cellular virus detection

Localization of RusV RNA on the cellular level was visualised via ISH using the RNAScope 2.5 HD Reagent Kit Red (Advanced Cell Diagnostics, USA) as previously described [18]. Brain tissue from a known RusV-positive animal served as positive control. BoDV-1 and TBEV antigen were detected on the cellular level via IHC. Two monospecific rabbit hyperimmune sera for the detection of BoDV-1 P (No. 130; dilution 1:4,000) and N (No. 201; dilution 1:4,000) were used as previously described [8]. Brain tissue from a BoDV-1-positive horse served as a positive control. TBEV antigen was detected using a polyclonal anti-TBEV rabbit serum (strain “Hochosterwitz”, dilution 1:2,000) [47, 48]. Reactions were performed as previously described [49] with the minor adjustment of using a secondary biotinylated goat anti-rabbit IgG antibody (1:200; Vector Laboratories, USA). Brain sections of an experimentally TBEV-infected mouse (kindly provided by Dr. Christina Puff, Department of Pathology, University of Veterinary Medicine Hannover) were included as positive control. Primary antibodies were replaced by irrelevant rabbit immunoglobulins (Bio-Genex, Fremont, USA) to test for specificity of the signals.

Slides were digitalised using a whole slide Aperio AT2 scanner (Leica, Germany) for BoDV-1 and TBEV cases and a whole slide Hamamatsu S60 scanner (Hamamatsu Photonics, K.K. Japan) for RusV cases. Chromogen signals were evaluated based on a previously published signal scoring protocol [6].

### Histopathology

Tissues from archived FFPE material were cut at 3 μm and routinely stained with haematoxylin and eosin. Inflammatory lesions were graded according to a previously published protocol [6].

## Results

### Retrospective detection of viral RNA by RT-qPCR in mammalian cases with lymphocytic meningoencephalitis

RusV was detected by RT-qPCR in one out of two maned wolves, two out of 50 cats and the only one donkey (Tables 1 and 2). With the exception of the fresh-frozen samples from the maned wolf, all positive samples had very high Cq values (> 35), presumably due to RNA degradation in the FFPE material (Table 2). BoDV-1 was detected in three out of eight horses with Cq values ranging from 21 to 28 (Tables 1 and 2). TBEV was detected only in one dog necropsied in 1995 (Cq 27; Tables 1 and 2). None of the other examined species tested positive for RusV, BoDV-1, or TBEV. IAV was not found in any of the samples tested. Host mRNA was detectable by RT-qPCR in all samples analysed confirming sufficient RNA quality.

**Table 1:** Case numbers of lymphocytic meningoencephalitis per species including numbers of positive cases

		Positive cases		
Species	Number of cases	RusV	BoDV-1	TBEV
Dog (*Canis lupus familiaris*)	78	0	0	1 (1.3%)
Cat (*Felis catus*)	50	2 (4%)	0	0
Cattle (*Bos taurus*)	12	0	0	0
Goat (*Capra aegagrus hircus*)	11	0	0	0
Horse (*Equus caballus*)	8	0	3 (37.5%)	0
Pig (*Sus scrofa domesticus*)	7	0	0	0
Sheep (*Ovis gmelini aries*)	6	0	0	0
Guinea pig (*Cavia porcellus*)	6	0	0	0
Ferret (*Mustela putorius furo*)	2	0	0	0
Maned wolf (*Chrysocyon brachyurus*)	2	1 (50%)	0	0
Donkey (*Equus asinus asinus*)	1	1 (100%)	0	0
Moose (*Alces alces*)	1	0	0	0
Striped hyena (*Hyaena hyaena*)	1	0	0	0
Raccoon (*Procyon lotor*)	1	0	0	0
Mink (*Mustela lutreola*)	1	0	0	0
Squirrel monkey (*Saimiri sciureus*)	1	0	0	0
Mouse (*Mus musculus f. domestica*)	1	0	0	0
Hamster (not specified)	1	0	0	0
Wombat (not specified)	1	0	0	0
total	191	4 (2.1%)	3 (1.6%)	1 (0.5%)

**Table 2:** Animals tested positive for viral neuropathogens in this study

Case ID	Species	Month and year of death	Detected viral pathogens	Material for PCR	RT-qPCR (C_q_ value)	ISH/IHC Score (6)	Sequencing (accession number)
Case #1	donkey	January 2017	RusV	FFPE	36.17	1	PV806687
Case #2	cat	January 2018	RusV	FFPE	39.73	2	PV806689
Case #3	cat	June 2018	RusV	FFPE	39.80	1	PV806688
Case #4	maned wolf	July 2024	RusV	Fresh frozen	25.74	2	PV806690
Case #5	horse	May 1996	BoDV-1	FFPE	27.11	1	n/a
Case #6	horse	December 2002	BoDV-1	FFPE	27.63	2	n/a
Case #7	horse	February 2005	BoDV-1	FFPE	21.66	2–3	PV806686
Case #8	dog	July 1995	TBEV	FFPE	27.20	1	n/a

*FFPE* formalin-fixed paraffin-embedded

### Virus sequence analysis and geographic distribution

**Fig. 1:**
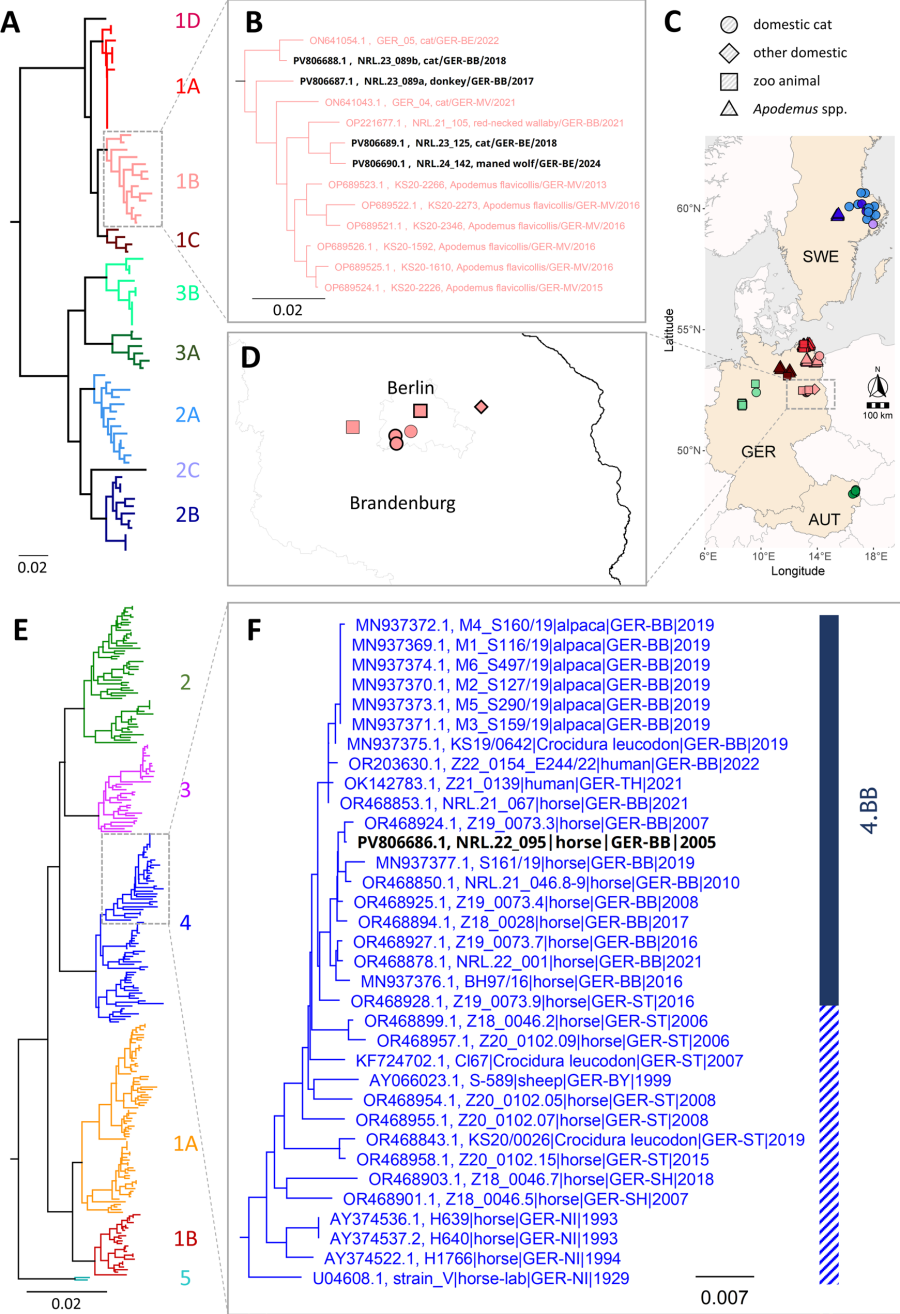
Phylogenetic analysis of RusV and BoDV-1 sequences. **A**, **E**: Neighbour-Joining trees were generated for the sequences determined in this study together with the sequences derived from public databases spanning the same sequence stretch (RusV: 409 nucleotides; *n* = 75; BoDV-1: 1,824 nucleotides, *n* = 260). A RusV clade 4 sequence (PP025855) from a mountain lion from Colorado, USA or BoDV-2 sequence No/98 (AJ311524) from a horse in Styria, Austria were used for rooting the trees (not shown). Colours represent RusV genotypes 1 A to 3B [50] or BoDV-1 clusters 1 A to 5 [10]. **B**, **F**: Subtrees containing the RusV or BoDV-1 sequences generated in this study (depicted in black). The dark blue vertical bar in panel F indicates BoDV-1 subclade 4.BB [10]. **C**, **D**: Geographic localization of RusV sequences. Colours represent RusV genotypes, as shown in panel **A**. Symbols of the four cases reported in this study are indicated by a thicker black line in panel **D**. AUT: Austria, GER: Germany, SWE: Sweden; BB: Brandenburg, BE: Berlin, BY: Bavaria; MV: Mecklenburg-Western Pomerania, NI: Lower Saxony, SH: Schleswig-Holstein, ST: Saxony Anhalt, TH: Thuringia.

The partial RusV nucleotide sequences generated from all four RusV-positive animals belonged to genotype 1B composed of nucleotide sequences from Berlin, Brandenburg, and Eastern Mecklenburg-Western Pomerania within the genetic clade 1 found in northeastern Germany, as defined by Pfaff et al. [50] (Fig. 1A-C). The RusV nucleotide sequence obtained from case #3 (cat; PV806688) had the highest genetic similarity to the sequence from a previously reported feline case from Berlin (ON641054; 99.3% nucleotide sequence identity; Fig. 1B) [6]. This cat originated from southwest of the border between Brandenburg and Berlin (Fig. 1D). The nucleotide sequences originating from case #2 (cat; PV806689) and case #4 (maned wolf; PV806690) were closely related to each other (99.3%) and to the sequence from an infected wallaby from a zoo located northeast of Berlin (OP221677; 98.3 to 98.5%; Fig. 1B) [18]. This cat was a stray animal, finally treated at the small animal clinic of Freie Universität Berlin. The maned wolf originated from a zoologic garden in the centre of Berlin (Fig. 1D). The nucleotide sequence from case #1 (donkey; PV806687), which was suspected to have been housed in Brandenburg approximately 20 km east of Berlin, was not particularly closely related to a specific other sequence within genotype 1B (max. 98.3%; Fig. 1B, D).

Generation of partial BoDV-1 nucleotide sequences from BoDV-1-positive horses was successful only for case #7, whereas it failed for cases #5 and #6, presumably due to highly degraded RNA in the FFPE samples. The sequence of case #7 (PV806686) belonged to the phylogenetic clade 4.BB within BoDV-1 cluster 4 (Fig. 1F). Clade 4.BB is composed almost exclusively of sequences originating from northwestern parts of Brandenburg [10]. The most closely related sequence originated from a horse from the district Ostprignitz-Ruppin in 2007 (OR468924; 99.8%; Fig. 1F). The exact origin of cases #7 und #6 remains undetermined, however, it is assumed that they originated from the federal state of Brandenburg. Case #5 most likely originated from the district Havelland in Brandenburg.

Attempts to generate partial TBEV sequences from case #8 yielded only a short sequence fragment of 81 nt length, which was not sufficient for phylogenetic analysis and submission to GenBank. However, BLAST analysis identified the sequence fragment to be identical to TBEV sequences from the Netherlands and the United Kingdom (accession numbers LC171402, MN661145, MZ969637, ON502378). In this case no information regarding the origin of the dog was available, other than its final clinical treatment in Berlin.

### Histologic lesions and cellular virus distribution

**Fig. 2:**
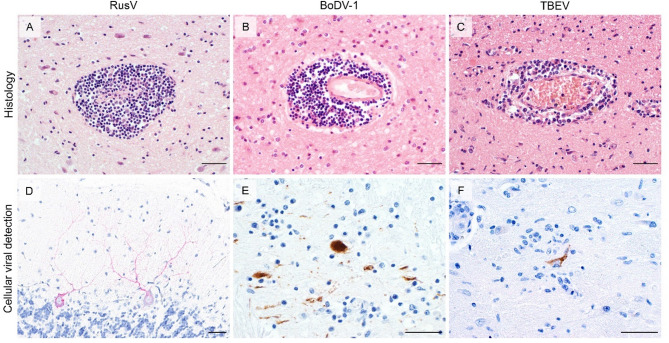
Inflammation and cellular localization of RusV, BoDV-1 and TBEV. **A-C**: Histopathology of the infected brains with perivascular cuffing, marked (**A** and **B**) and moderate (haematoxylin and eosin stain). **D-F**: Representative cellular localization via RNA in situ hybridization for RusV or immunohistochemistry for BoDv-1 and TBEV in neurons **D**: fast red as chromogenic labelling, **E** and **F**: 3,3′-Diaminobenzidin (DAB) for chromogenic labelling, all: Mayer’s haematoxylin counter stain). Bars represent 40 μm.

In all cases, histopathology revealed lymphocytic meningoencephalitis with perivascular lymphocytic cuffing with or without nodular glial cell proliferation representing grades from mild (cases #1, #4, #7), moderate (cases #2, #6, #8) to severe (case #3 and #5) (Table 2; Fig. 2A-C).

Virus-specific ISH or IHC confirmed respective viral infections in all cases in which virus was detected by RT-qPCR. Each of the viruses was located predominantly in neurons.

For RusV, the distribution of specific granular chromogen signals obtained by ISH in neurons was consistent with previous reports [6]. Positive cells were predominately found in the gray matter of the cerebrum, especially the hippocampus, as well as the cerebellum, particularly in Purkinje cells (Fig. 2D). Infected neurons, however, were only occasionally spatially associated with a corresponding inflammation (Fig. 2D).

In all three equine Borna disease (BD) cases, BoDV-1 N and P proteins were localised via IHC to neuronal perikaryal and axonal structures throughout the neuropil with a varying patchy to diffuse distribution throughout all analysed brain structures including neocortex and cerebellum, consistent with previous observations [28] (Fig. 2E). BoDV-1-infected cells were more often directly associated with inflammation, as compared to the RusV cases (Fig. 2E).

TBEV antigen was detected by IHC in the cytoplasm of single neurons scattered throughout the cerebrum and cerebellum (Fig. 2C). Overall, low numbers of TBEV-positive neurons were affected by perineuronal satellitosis (Fig. 2F).

### Case histories, clinical data and gross pathology

Case histories, clinical presentation,; and necropsy findings are summarised for all cases in Additional file 2: Table S2. For all animals, neurological signs and sometimes behavioural alterations had been reported with an acute to subacute course of disease. The time from the reported onset of clinical signs to euthanasia due to unfavourable prognoses ranged from one day to two weeks (Additional file 2: Table S2).

## Discussion

Our cross-species analysis of selected viral neuropathogens detected the recently discovered RusV in three different species. The maned wolf, a zoo animal species, is a newly identified RusV-susceptible host, thereby extending the host range of RusV to the Canidae family. Dogs, on the other hand, were not found to be infected by RusV in this study. Notably, the virus had first been discovered in a zoo in northern Germany and RusV-induced encephalitis had initially been detected mainly in zoo animals [4, 5, 7, 18, 19]. One likely explanation for this could be the high percentage of zoo animals which are submitted for pathological examination as part of the health monitoring in zoos.

In addition, RusV was found in two cats and one donkey. These species have already been confirmed to be susceptible hosts [4, 6]. In cats, RusV has been predominately found to be associated with staggering disease [6, 22, 23]. The condition is characterised by a lymphocytic meningoencephalitis that typically manifests as hindlimb ataxia and is very well known in Sweden and Austria [51–53]. Additionally to these two countries, the virus had previously also been detected in cats from Germany with lymphocytic meningoencephalitis [6]. In previously investigated cases from Sweden, Austria, and Germany, 27 out of 29 cases (93.1%) from all three countries [6], seven out of 23 cases (30.4%) from Austria [22] or 13 out of 14 cases (92.9%) from Sweden [23] were positively tested for RusV. Taken into account the previously published case of a cat from Berlin [6], only 5.9% of cats diagnosed with lymphocytic meningoencephalitis in Berlin and Brandenburg were positive for RusV. The higher detection rates in the previous studies might be attributed to stricter inclusion criteria focusing on the typical histopathological and clinical presentation of staggering disease and the fact that samples were mainly from hotspot regions of this neurological disease.

Viral sequences generated from the cases from Sweden, Austria, and Germany exhibit phylogenetic differences and can be classified in three main clades, which mostly align with the geographical locations. The sequences generated in this study belong to genotype 1B, which has been found in Berlin, Brandenburg, and parts of the neighbouring federal state Mecklenburg-Western Pomerania. Data from Sweden suggest that RusV infections have been occurring for decades, dating back to the 1970 s and continuing up until at least 2021 [6, 23]. In contrast, a retrospective study from Austria revealed that RusV-positive cases had only been found during a limited time frame, mainly in the 1990 s [6, 22]. In our current study, RusV infections in the geographical area around Berlin were detected between 2017 (case #1, donkey) and 2024 (case #4, maned wolf). The reason for the epidemiolocal discrepancy between the different regions is unclear. Continuous monitoring of RusV infection is thus recommended and awareness for the clinical and pathological findings of RusV infections is warranted.

To date, yellow-necked field mice are considered to be a putative reservoir for RusV in northeastern Germany [4, 7, 24]. The close genetic relationship between the generated viral sequences from this study and sequences from the same area found in previous studies [6, 18] indicates an endemic presence of the virus in the Berlin-Brandenburg region and local infection sources of the tested animals. Unfortunately, data pertaining to RusV-infected yellow-necked field mice and other rodents from Berlin and most of Brandenburg are still lacking. All four RusV-infected animals in this study had access to the outdoors, which has been suggested as a risk factor for staggering disease in Sweden [6]. This supports the hypothesis of a putative RusV spillover from a rodent reservoir, although the route of transmission remains unclear.

BoDV-1, the agent of the zoonotic BD, could only be identified in horses in this study, with a detection rate of 37.5%. Horses, in addition to sheep, New World camelids, and humans, are well-documented spillover hosts for BoDV-1 [10, 11, 28, 54]. In contrast, other species such as dogs, cats, a pigmy hippopotamus, a Eurasian beaver, cattle, and rabbits have only sporadically been reported to be susceptible to this virus [10, 55–59]. This study underlines the predominant spectrum of susceptible BoDV-1 species.

BoDV-1-endemic areas are restricted to Germany, Austria, Switzerland, and Liechtenstein, and BoDV-1 sequences have been shown to possess remarkable geographic associations, with individual phylogenetic clades occupying distinct areas [8, 10, 11, 31, 32]. While northwestern Brandenburg is known to be an endemic area for BoDV-1, no cases have been described from the eastern and southern parts of this federal state or from Berlin [10]. Unfortunately, in these archived BoDV-1-positive cases, the specific locations of the horses’ stables could no longer be determined. Information on the owners’ residences and the fact that all animals were finally treated in the equine clinic of the Freie Universität Berlin suggests that they were stabled and probably infected in the greater Berlin-Brandenburg region. This is further underlined by the BoDV-1 sequence generated from one horse in this study (case #7), which belongs to clade 4.BB, composed of sequences from shrews, domestic mammals, and humans originating from northwestern Brandenburg [10].

TBEV was only detected in one dog from 1995 in this study. The low number of TBEV-positive cases is in line with other sporadic case reports of fatal TBEV infection in dogs [9, 34, 60]. Overall, clinical disease due to TBEV or even a fatal outcome seems to be rare in dogs [61]. TBEV occurs in several European and Asian countries [62]. It should be noted, that only parts of southern Brandenburg were recently classified as TBEV risk areas towards the very end of the study period [63, 64] and the virus was only considered endemic in Brandenburg in the 1960 s [65]. This may also have contributed to the low number of cases. The TBEV-positive dog found in this study was finally treated at the small animal clinic of the Freie Universität Berlin. Unfortunately, neither the owners’ residence nor travel information of the dog prior to onset of neurological signs were available. During the 1990 s, TBEV-endemic areas were particularly identified in southern Germany (Baden-Württemberg and Bavaria) and Hesse. No autochthonous human infections were reported at that time in Berlin-Brandenburg [65]. For this reason, it seems unlikely that the dog had been infected in Berlin-Brandenburg. Unfortunately, determination of sequence information sufficient to further analyse the potential origin of the virus was unsuccessful, likely due to the highly degraded RNA in the archived sample.

Clinical findings, histopathology features, and distribution of viral RNA or antigen in the cases examined in this study were consistent with those reported in previous research on these three viruses [4, 6–9, 18, 19, 21, 22, 28, 32, 34, 66].

In contrast to the other three viruses, IAV, such as HPAIV H5, was not detected in any of the samples investigated here. This may appear somewhat unexpected as spillover events to mammals have been repeatedly documented in the country after the first arrival of early clade gs/GD HPAIV H5 in the winter of 2006 [67] and more recent clades such as 2.3.4.4b [15, 16, 68, 69]. Of note, our cohort even included scavenging and predatory mammals which seem to be at high risk of infection following alimentary exposure or direct contact with HPAIV H5 infected birds [70].

This study has potential limitations. Our screening approach used spatial tissue detection of the viruses via IHC or ISH only to confirm positive cases identified by RT-qPCR and not as an additional screening method. Since the sensitivity of RT-qPCR from FFPE material may be reduced due to RNA degradation [6, 10], we cannot exclude that we missed cases that may have been detectable by IHC or ISH. As samples with known other causes of encephalitis were excluded, putative co-infections with the analysed viruses cannot be ruled out. This may have resulted in an underestimation of infected cases here. However, at least for RusV no such co-infections have been found in previous studies [4, 7, 18]. Still, future studies should clarify whether co-infections or even non-infectious co-morbidities may affect the course of the diseases caused by these four viruses. Moreover, thorough systematic prevalence studies on spillover infections with the four viruses analysed here seem unavailable. Rather, there are only few limited reports in selected subpopulations [6, 10, 12, 22, 23, 71].

## Conclusions

Spillover infections occur for the viral neuropathogens RusV, BoDV-1, and TBEV in certain species. The maned wolf was identified as a new susceptible species for RusV. This identification of RusV infection in the Canidae family further extends the known host range of the virus. This finding underscores the importance of not excluding any species from diagnostic testing in the future. RusV and BoDV-1 were in particular detected in cats and horses, respectively. An infection with TBEV is rarely associated with fatal meningoencephalitis in dogs. RusV and BoDV-1 remain a potential concern in northeastern Germany, requiring ongoing surveillance to better understand their clinical and geographic dynamics.

## Supplementary Information

Supplementary Material 1.

Supplementary Material 2.

## Data Availability

The datasets used and/or analysed during the current study are available from the corresponding author on reasonable request. Sequence data that support the findings of this study have been deposited at GenBank with the primary accession codes: PV806686, PV806687, PV806688, PV806689, PV806690.

## Abbreviations

RNA: Ribonucleic acid
RT: qPCR-reverse transcription quantitative polymerase chain reaction
RusV: Rustrela virus
BoDV-1: Borna disease virus 1
BD: Borna disease
TBEV: Tick-borne encephalitis virus
TBE: Tick-borne encephalitis
IAV: Influenza A virus
HPAIV H5: Highly pathogenic avian influenza virus of hemagglutinin subtype H5
ISH: In situ hybridisation
IHC: Immunohistochemistry
FFPE: Formalin-fixed, paraffin-embedded
Cq: Cycle of quantification
