# Supplementary material for: Spillover infections by rustrela virus, borna disease virus 1 and tick-borne encephalitis virus revealed by retrospective screening of mammalian encephalitis of unknown origin
Source: BMC Vet Res. 2025 Nov 15;21:671. doi: 10.1186/s12917-025-05132-w (PMC12619152; doi:10.1186/s12917-025-05132-w)
Supplement: Supplementary file 1 — Supplementary Material 1. [file 12917_2025_5132_MOESM1_ESM.pdf]

Supplementary material:

Table S1: Primer, probes, and cycler protocols for detection of viral neuropathogens

Assay	Primer/Probe name	Sequence 5' to 3'	Cycler set up	Time	Temperature	Cycles	Reference
panRusV-2	RusV_234+	CCCCGTGTTCCTAGGCAC	Activation Taq	10 min	95 °C		modified according to
	RusV_323-	TCGCCCCATTCWACCCAATT	Denaturation	15 s	95 °C	45	Thilén et al., 2024
	RusV_257_P	FAM-TGAGCGACCACCCAGCACTCCA-BHQ1	Annealing	30 s	60 °C		
			Elongation	30 s	72 °C		
BoDV-1-P	BoDV-1_1303+	TCCCTGGAGGACGAAGAAGAT	Reverse Transcription	10 min	50 °C		Schindler et al., 2007
	BoDV-1_1371-	CTTCCGTGGTCTTGGTGACC	Activation Taq	1 min	95 °C		
	BoDV-1_1326-P	FAM-CCAGACACTACGACGGGAACGA-BHQ1	Denaturation	10 s	95 °C	45	
			Annealing	30 s	57 °C		
			Elongation	30 s	68 °C		
TBEV1_Mix	F-TBE 1	GGG CGG TTC TTG TTC TCC	Reverse Transcription	10 min	45 °C		Schwaiger & Cassinotti,
	R-TBE 1	ACA CAT CAC CTC CTT GTC AGA CT	Inactivation/Activation	9 min	95 °C		2003
	TBE-FAM-WT	FMA-TGA GCC ACC ATC ACC CAG ACA CA-BHQ1	Denaturation	15 s	95 °C	50	
			Annealing/Elongation	60 s	58 °C		
Pan AI assay	M1-F	AGA TGA GYC TTC TAA CCG AGG TCG	Reverse Transcription	10 min	45 °C		Hassan et al., 2022
	M1-FAM	FAM-TCA GGC CCC CTC AAA GCC GA-BHQ1	Activation Taq	10 min	95 °C		
	M1-R1	TGC AAA AAC ATC TTC AAG TYT CTG	Denaturation	15 s	95 °C		
	M1-R2	TGC AAA GAC ACT TTC CAG TCT CTG	Annealing	20 s	56 °C	45	
	M1-R3	TGC AAA I(Inosine)AC ATC YTC AAG TYT CTG	Elongation	30 s	72 °C		
EF1alpha	Primer (upstream)	CAAAAACGACCCACCAATGG	Activation Taq	10 min	95 °C		Braun et al., 2010
	Primer (downstream)	GGCCTGGATGGTTCAGGATA	Denaturation	15 s	95 °C		
	TaqMan Probe	FAM-AGCAGCTGGCTTCACGCTCAGGTG-BHQ1	Annealing	30 s	60 °C	45	
			Elongation	30 s	72 °C		
EGFP-Mix 1	EGFP1-F	GAC CAC TAC CAG CAG AAC AC	Reverse Transcription	10 min	45 °C		Hoffmann et al., 2006
	EGFP-2-R	GAA CTC CAG CAG GAC CAT G	Inactivation/Activation	9 min	95 °C		
	EGFP- Probe 1-HEX	HEX-AGC ACC CAG TCC GCC CTG AGC A-BHQ1	Denaturation	15 s	95 °C	50	
			Annealing/Elongation	60 s	58 °C		
ß-Actin-Mix2	ACT-1005-F	CAGCACAATGAAGATCAAGATCATC	Reverse Transcription	10 min	45 °C		Toussaint et al., 2007
	ACT-1135-R	CGGACTCATCGTACTCCTGCTT	Activation Taq	2 min	95 °C		
	ACT-1081- HEX	HEX-TCGCTGTCCACCTTCCAGCAGATGT-BHQ1	Denaturation	5 s	95 °C	45	
			Annealing/Elongation	20 s	60 °C		
