# Supplementary material for: Spillover infections by rustrela virus, borna disease virus 1 and tick-borne encephalitis virus revealed by retrospective screening of mammalian encephalitis of unknown origin
Source: BMC Vet Res. 2025 Nov 15;21:671. doi: 10.1186/s12917-025-05132-w (PMC12619152; doi:10.1186/s12917-025-05132-w)
Supplement: Supplementary file 2 — Supplementary Material 2. [file 12917_2025_5132_MOESM2_ESM.pdf]

Table S2: Case histories, clinical and pathological findings

Case ID	Agent	Species	Sex	Age in years	Case history and clinical signs	Necropsy findings*	Histology of brain (including grade (9))	Histology of other organs
Case 1	RusV	donkey	female	ca. 3	progressive weakness<br>2 days later:<br>recumbency; trismus,<br>recurring, horizontal nystagmus<br>reduced threat response,<br>reduced pupillary reflex,<br>epileptoid seizures<br>euthanasia 4 days after onset of clinical signs	no specific findings	mild, multifocal, lymphoplasmacytic meningoencephalitis	lung: mild, multifocal, lymphoplasmacytic infiltrates;<br>liver: mild, multifocal, lymphoplasmacytic infiltrates;<br>kidney: mild, multifocal lymphoplasmacytic pyelonephritis;<br>ganglion coeliacum: minimal lymphocytic infiltration
Case 2	RusV	cat	male	unknown	stray animal,<br>suspicion of a neurological process<br>euthanasia 3 days after admission to the clinic	mild dehydration,<br>thoracic cavity: 10 ml of serosanguineous fluid,	moderate, multifocal, lymphoplasmacytic meningoencephalitis	lung: severe, focal, chronic, granulomatous vasculitis<br>(suspicion of chronic partial embolic obstruction)
Case 3	RusV	cat	female	1	pregnant, ataxia, tachypnoea<br>3 days later:<br>caesarean section: delivery of four living fetuses (ca. 70 g), (one kitten still alive)<br>seizure after anesthesia, aggressive behavior,<br>1 day later: head tremor<br>euthanasia 1 week after onset of clinical signs	mild dehydration,<br>nasal cavity:<br>severe, diffuse, chronic-active necrosuppurative rhinitis with severe atrophy of conchae;<br>cerebellum: protrusion of vermis cerebelli into the foramen magnum	severe, multifocal, lymphoplasmacytic meningoencephalitis	mammary gland: active
Case 4	RusV	maned wolf	female	1.5	unsteady gait, treatment with vitamin B complex, antibiotics, antiprotozoal agent<br>progression to ataxia, proprioceptive deficits in hindlimbs, dilated pupils;<br>7 days later: no feed intake, recumbency;<br>treatment: infusion, dexamethasone<br>clinical improvement with intravenous medication;<br>2 weeks after start of signs, marked clinical deterioration;<br>euthanasia 17 days after onset of clinical signs	skin shoulder + hip:<br>severe, multifocal, acute, necrosuppurative dermatitis	mild, multifocal, lymphoplasmacytic meningoencephalitis	lung: moderate, subacute, alveolar and interstitial suppurative bronchopneumonia;<br>kidneys: mild, chronic, lymphocytic pyelitis
Case 5	BoDV-1	horse	male, neutered	4	suspicion of colic,<br>reduced feed and water uptake,<br>admission to clinic: stall walking, unsteady gait, backhand weakness;<br>4 days later:<br>discharge from clinic against medical advice,	no specific findings	severe, multifocal, lymphoplasmacytic encephalitis	spinal cord: moderate lymphoplasmacytic myelitis;<br>trigeminal ganglion: moderate lymphoplasmacytic ganglionitis,<br>optical nerve: mild lymphoplasmacytic perineuritis;

					euthanasia due to increasing somnolence and backhand weakness 1 week after onset of clinical signs			liver: centrilobular degeneration and liver cell necrosis, chronic congestion; heart: acute myocardial degeneration; kidney: chronic, interstitial nephritis
Case 6	BoDV-1	horse	female	5	neurological signs: stall walking, proprioception disorder, euthanasia around 2 weeks after onset of clinical sings	stomach: gastrophilosis	moderate, multifocal, lymphoplasmacytic encephalitis	spinal cord: mild lymphoplasmacytic myelitis; heart: occasional, acute myocardial necrosis; lung: chronic, suppurative bronchitis
Case 7	BoDV-1	horse	female	2.5	depression, circular movement, tilted head, trismus, ventroflexion; guttural pouch: swelling, serous to hemorrhagic fluid; avulsion of right *Musculus capitis longus*, euthanasia 1 week after onset of clinical signs	head: diffuse edema	mild, multifocal, lymphoplasmacytic encephalitis; subdural and leptomeningeal, acute hemorrhages	heart: myocardial fibrosis; guttural pouch: edema, hemorrhage
Case 8	TBEV	dog	male	9.5	long term treatment: diabetes mellitus, Cushing syndrome; easily startled, restless, neurological signs including stall walking, nystagmus; euthanasia 1 day after onset of neurological sings	heart: fibrosis of the mitral valve, hypertrophy and dilation of the left ventricle and atrium	moderate, multifocal, lymphoplasmacytic encephalitis	kidney: chronic, interstitial, fibrosing nephritis; pancreas: islet atrophy; adrenal gland: multiple hyperplasia liver: hepatolipidosis

*****Agonal related findings are not listed.
